# Cloning and functional characterization of the peptide deformylase encoding gene *EuPDF1B* from *Eucommia ulmoides* Oliv

**DOI:** 10.1038/s41598-024-62512-2

**Published:** 2024-05-21

**Authors:** Yumei Wang, De-Gang Zhao

**Affiliations:** 1 Key Laboratory of Plant Resources Conservation and Germplasm Innovation in Mountainous Region (Ministry of Education), College of Life Sciences, Institute of Agro-Bioengineering, Guizhou University, Guiyang, 550025 China; 2 Plant Conservation Technology Center, Guizhou Key Laboratory of Agricultural Biotechnology, Guizhou Academy of Agricultural Sciences, Guiyang, 550006 China

**Keywords:** Peptide deformylase, Promoter, Transgenic plants, Biological techniques, Biotechnology, Cell biology, Genetics, Molecular biology, Physiology, Plant sciences

## Abstract

Peptide deformylase can catalyse the removal of formyl groups from the N-terminal formyl methionine of the primary polypeptide chain. The peptide deformylase genes of a few herbaceous plants have been studied to some extent, but the peptide deformylase genes of woody plants have not been studied. In this study, we isolated *EuPDF1B* from *Eucommia ulmoides* Oliv. The full-length sequence of *EuPDF1B* is 1176 bp long with a poly-A tail and contains an open reading frame of 831 bp that encodes a protein of 276 amino acids. EuPDF1B was localized to the chloroplast. qRT‒PCR analysis revealed that this gene was expressed in almost all tissues tested but mainly in mature leaves. Moreover, the expression of *EuPDF1B* was enhanced by ABA, MeJA and GA and inhibited by shading treatment. The expression pattern of *EuPDF1B* was further confirmed in EuPDF1Bp: GUS transgenic tobacco plants. Among all the transgenic tobacco plants, EuPDF1Bp-3 showed the highest GUS histochemical staining and activity in different tissues. This difference may be related to the presence of enhancer elements in the region from − 891 bp to − 236 bp of the *EuPDF1B* promoter. In addition, the expression of the chloroplast gene *psbA* and the net photosynthetic rate, fresh weight and height of tobacco plants overexpressing *EuPDF1B* were greater than those of the wild-type tobacco plants, suggesting that *EuPDF1B* may promote the growth of transgenic tobacco plants. This is the first time that *PDF* and its promoter have been cloned from woody plants, laying a foundation for further analysis of the function of *PDF* and the regulation of its expression.

## Introduction

Protein synthesis in the cytoplasm begins with methionine, whereas protein synthesis in chloroplasts and mitochondria begins with N-formylmethionine. However, most mature proteins do not retain their initial N-terminal methionine (methionine in the cytoplasm and N-formylmethionine in the organelles), suggesting that N-formylmethionine is excised during the maturation of most proteins. N-terminal methionine excision is a ubiquitous protein modification^1^. This modification process in plants involves special mechanisms, and in addition to methionine aminopeptidase, peptide deformylase (PDF) has also been shown to be involved in the removal of N-formylmethionine^2^. PDF can catalyse the removal of formyl groups from the N-terminal formylmethionine of the primary polypeptide chain^3^ and has been shown to be widespread in eukaryotes^2,4,5^. In bacteria, the removal of N-terminal formyl groups is a prerequisite for subsequent N-terminal modifications, such as *N*-acetylation, *N*-methylation, and *O*-phosphorylation^6–8^.

Actinonin is a pseudopeptide with an N-terminal methionine analogue^9^ and the related N-formylhydroxylamine derivative BB-3497^10^. This small molecule can bind very tightly to the active site of PDF, thus greatly weakening the deformation reaction^11–13^. Actinonin is the most potent PDF inhibitor found thus far, and it has been shown to specifically inhibit PDF in plants^14^ as well as bacteria^9^. Therefore, researchers have used actinonin to study the role of PDF in plant organelles. It has been shown that actinonin can reversibly bind to the active site of bacterial PDF with nanomolar affinity, resulting in the retention of formylmethionine on nascent proteins in bacterial cells^13,15,16^. Inhibition of PDF activity in bacterial cells with actinonin rapidly induces cellular stress responses, particularly those related to protein misfolding and membrane defects, followed by global downregulation of metabolic pathways^17^. These results suggest that PDF plays a crucial role in maintaining the normal life activities of bacteria.

Eukaryotic peptide deformylases are also sensitive to actinonin^14,18^. Exposure of *Arabidopsis thaliana* (*A. thaliana*) to actinonin resulted in leaf bleaching and severe growth retardation and had significant dose-dependent effects^18^. After high concentrations of actinonin were applied to pea leaves, the fresh weight and dry weight of the pea plants decreased significantly, and the leaves exhibited an albino phenotype^18^. Exposure of *Chlamydomonas reinhardtii* to actinonin led to rapid degradation of all the newly synthesized photosystem II (PSII) complex-associated proteins^19^. After actinonin treatment of tobacco plants, D1 protein accumulation in PSII components decreases, PSII decomposes, and eventually leads to leaf death^20^. Transmission electron microscopy revealed that the chloroplasts and mitochondria of a PDF1B-deficient rice mutant were seriously damaged and that the mitochondria were slightly altered^21^.

To date, PDF has been extensively studied only in prokaryotes and, to a lesser extent, in a few herbaceous plants, such as *A. thaliana*, rice, and tobacco. However, *PDF* genes in woody plants have not been studied. *Eucommia ulmoides* Oliv (*E. ulmoides*) is not only an important traditional medicinal plant in China but also a high-quality gum source plant. With the exploration of its medicinal and gum value, this plant has received increasing attention from researchers. In this study, we isolated the *EuPDF1B* promoter and gene from *E. ulmoides* and characterized its specificity and induced expression pattern. Moreover, its function was analysed in transgenic tobacco overexpression lines.

## Results

### Isolation and identification of the *EuPDF1B*

**Figure 1:**
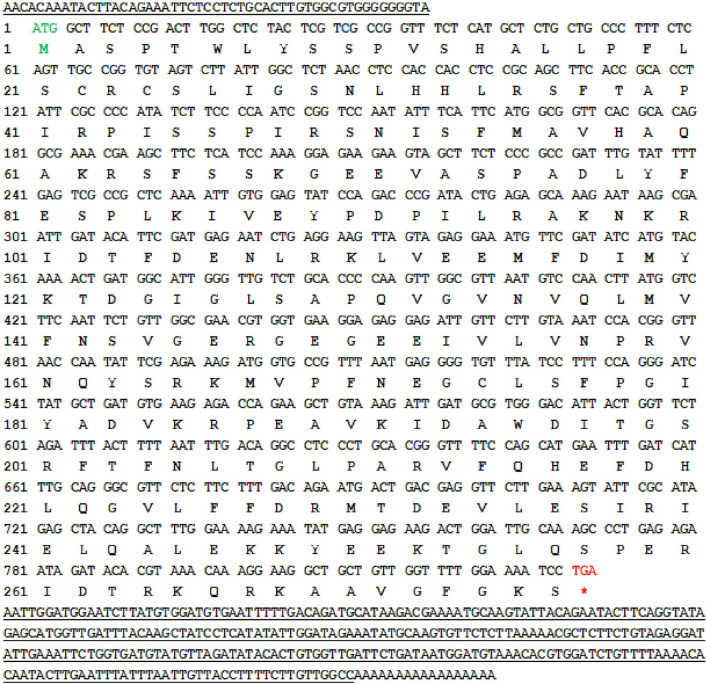
Nucleotide and deduced amino acid sequence of the *EuPDF1B* from *E. ulmoides*. The underlined section in front of the ATG indicates the 5’ end untranslated regions. The underlined portion after TGA indicates the 3’ end untranslated regions.

**Figure 2:**
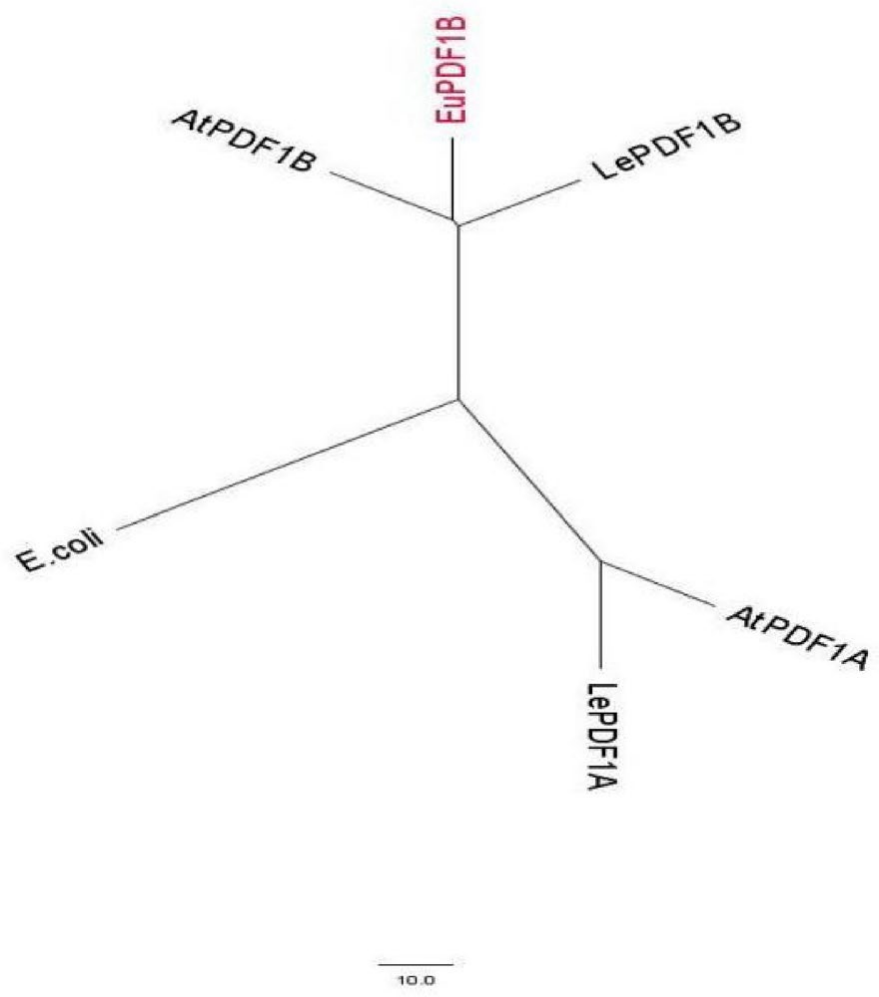
Phylogenetic tree of PDFs from *E. coli*, *A. thaliana* (AtPDF1A and AtPDF1B), *S. lycopersicum* (LePDF1A and LePDF1B) and *E. ulmoides* (EuPDF1B). The scale corresponds to 10 amino acid residues.

The full-length sequence of *EuPDF1B* is 1176 bp in length with a poly-A tail and contains an open reading frame of 831 bp that encodes a protein of 276 amino acids (Fig. 1). The CD-search tool in NCBI was used to predict the conserved domains of EuPDF1B, and the results showed that the protein has three absolutely conserved and unique motifs, which together constitute the entire active site of PDF. The amino acid sequence of this protein was compared with PDF amino acid sequences of other species, including *Escherichia Coli* (*E. Coli*) (accession no. CAA54826.1), *A. thaliana* (accession no. AF250959.1 and AF269165.1) and *Solanum lycopersicum* (*S. lycopersicum*) (accession no. AF250958.1 and AF271258.1), and an evolutionary tree was constructed using the neighbour-joining method in MEGA version 2.1. The results showed that the protein can be grouped with AtPDF1B and LePDF1B in the phylogenetic tree (Fig. 2). The above information confirmed that the cloned gene was indeed *PDF1B* and was named *EuPDF1B*.

### Expression patterns of *EuPDF1B* in *E. ulmoides*

**Figure 3:**
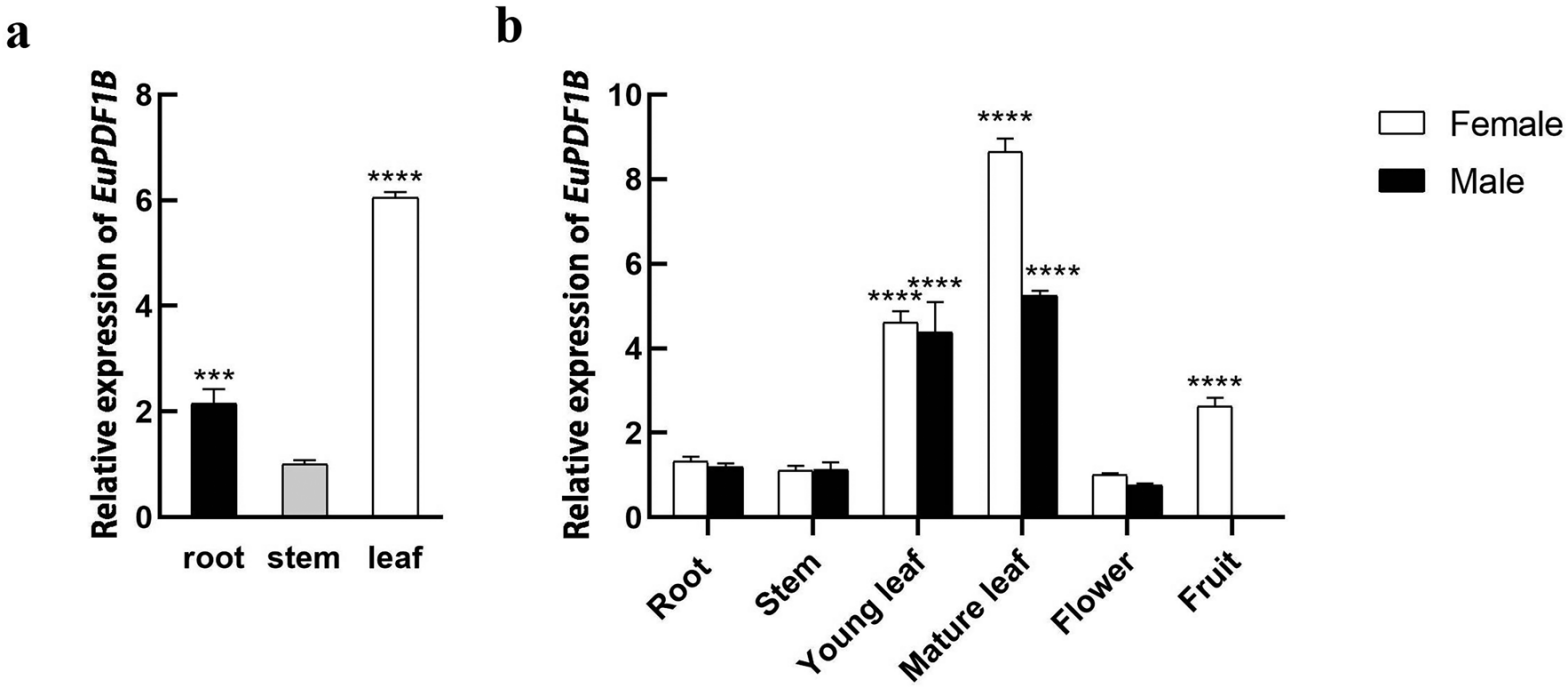
Expression patterns of *EuPDF1B* in *E. ulmoides.* **a** Relative expression levels of *EuPDF1B* in *E. ulmoides* seedlings. **b** Relative expression levels of *EuPDF1B* in *E. ulmoides* male and female plants. The error bars represent standard deviations of three replicates, ***P < 0.001, ****P < *0.0001*.

The expression level of *EuPDF1B* in different tissues of *E. ulmoides* seedlings and male and female plants was analysed by qRT‒PCR. The results showed that the expression of *EuPDF1B* in the leaves was significantly greater than that in the other tissues. In the seedlings, the expression level of *EuPDF1B* in the leaves was 3 times that in the roots and 6 times that in the stems (Fig. 3a). In male and female plants, the expression level of *EuPDF1B* in mature leaves was also significantly greater than that in other parts (Fig. 3b). This finding is consistent with the finding that the expression of *PDF1B* in mature leaves of rice is greater than that in young leaves^22^. Since leaves are the main organs involved in photosynthesis in plants and *EuPDF1B* is expressed mainly in leaves, it is speculated that EuPDF1B may play an important role in maintaining normal photosynthesis in plants.

### Subcellular localization analysis of EuPDF1B

**Figure 4:**
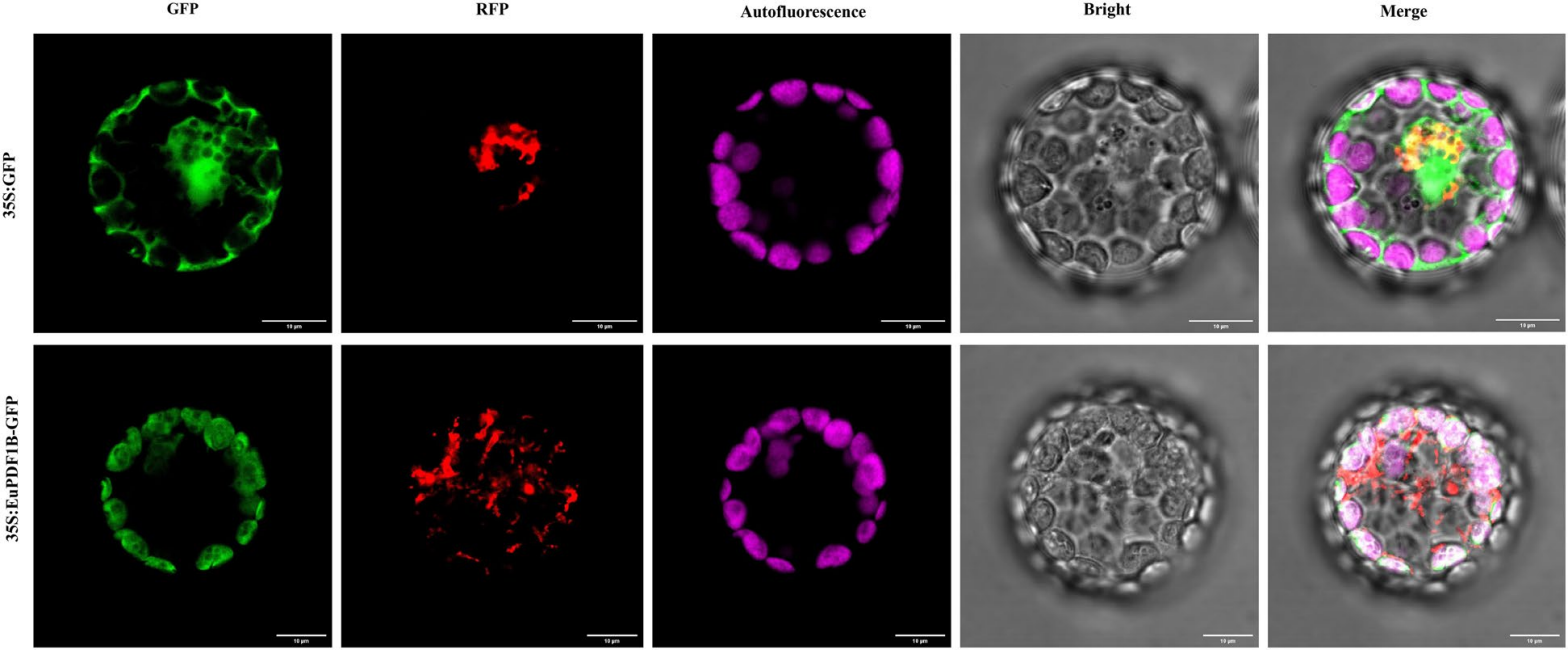
Subcellular localization of EuPDF1B. Scale bars = 10 μm.

Subcellular localization revealed that the positions of chloroplast autofluorescence and green fluorescence from EuPDF1B-GFP overlapped, while the positions of green fluorescence and red fluorescence did not overlap (Fig. 4). These results indicate that EuPDF1B is located in chloroplasts but not in mitochondria, in contrast to the finding that PDF1B in rice and *S. lycopersicum* is located in both chloroplasts and mitochondria^22^; the specific reasons for this difference need to be further studied. EuPDF1B is located in chloroplasts, which is consistent with the finding that the expression level of *EuPDF1B* in leaves is significantly greater than that in other parts, suggesting that EuPDF1B may play an important role in the maintenance of plant photosynthesis.

### Overexpression of *EuPDF1B* promotes tobacco plant growth

**Figure 5:**
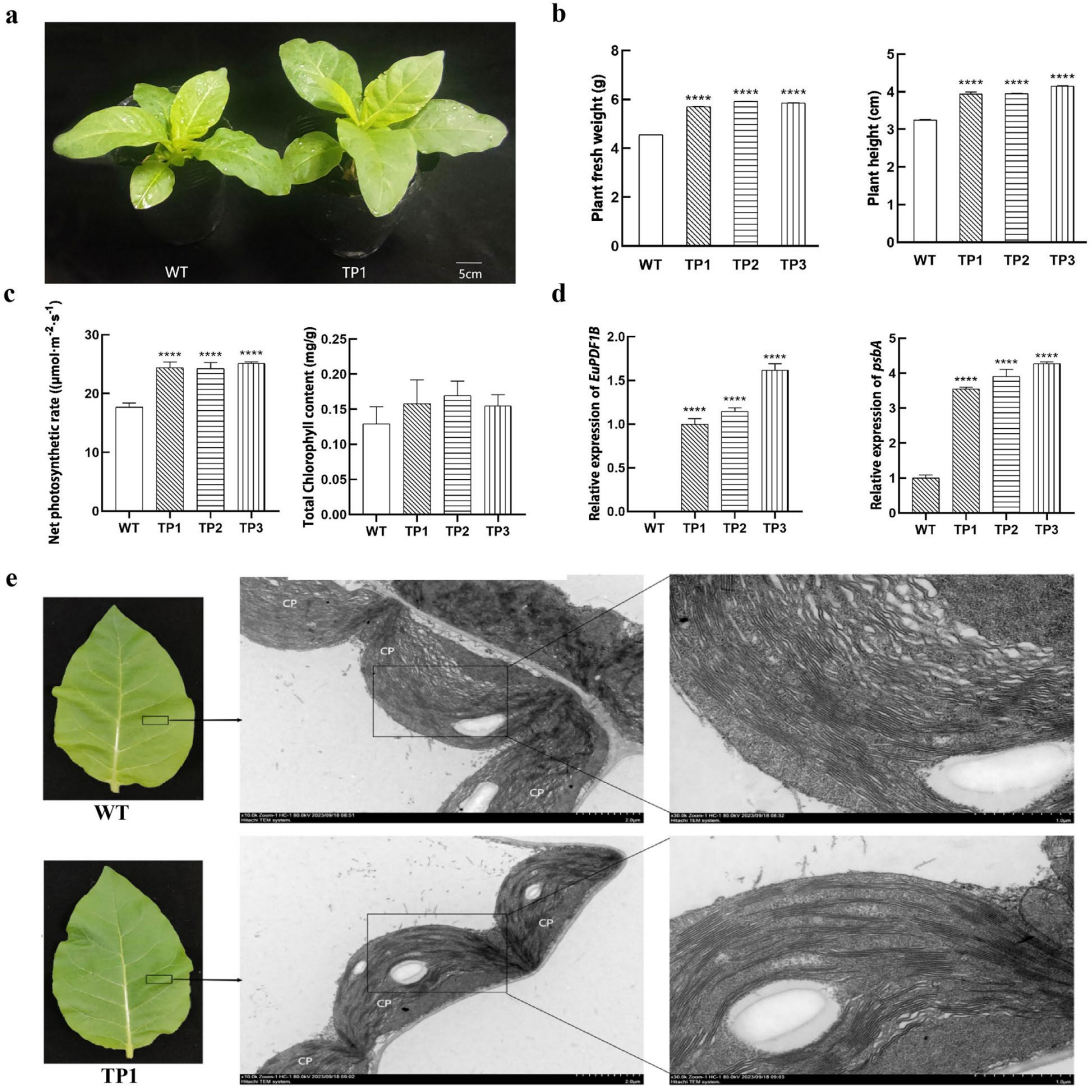
Characterization of the tobacco *EuPDF1B* line. **a** One-month-old wild-type and *EuPDF1B* transgenic plants. **b** Phenotypic indicators. **c** Physiological indexes. **d** Relative expression levels of genes. **e** TEM observation of chloroplasts. WT: wild-type tobacco plants, TP: transgenic tobacco plants. The error bars represent standard deviations of three replicates, ****P < *0.0001*. Cp indicates chloroplasts.

Transgenic tobacco plants overexpressing *EuPDF1B* were identified by GUS staining and PCR and three plant lines (TP1, TP2, TP3) with high expression level of *EuPDF1B* were selected for subsequent experimental study. Compared with those of wild-type tobacco plants and transgenic tobacco plants cultured under the same conditions, the average plant height, fresh weight and net photosynthetic rate of the transgenic tobacco plants were significantly greater than those of wild-type tobacco plants, but there was no significant difference in total chlorophyll content (Fig. 5a–c). These results indicated that the overexpression of *EuPDF1B* promoted the growth of tobacco plants. Previous studies have reported that silencing *PDF1B* can affect the expression of chloroplast coding genes. Considering that the D1 protein in PSII is the preferred substrate for *PDF1B* deformylation^23^ and is also encoded by chloroplast genes, we examined the expression of the *psbA* gene, which encodes the D1 protein, in transgenic tobacco. The expression level of *psbA* in the transgenic tobacco plants was 4 times greater than that in the wild-type tobacco plants (Fig. 5d), indicating that the overexpression of *EuPDF1B* promoted the expression of *psbA* in tobacco plants. Moreover, the average number of chloroplasts in each mesophyll cell of the transgenic tobacco plants was 4 more than that in the wild-type tobacco plants, but the difference was not significant. However, the chloroplast grana thylakoid membranes of the transgenic tobacco plants were more densely arranged than those of the wild-type tobacco plants (Fig. 5e).

### Isolation and *cis*-element analysis of the *EuPDF1B* promoter

In this study, we cloned the 1714 bp promoter region of the *EuPDF1B* gene. The PlantCARE online website was used to analyse and predict the *cis*-acting elements in the promoter sequence. The results showed that the promoter region contained core promoter elements such as the CAAT box and TATA boxes as well as *cis*-acting elements related to the light response and hormone response, including 1 GT1 motif, 2 G-boxes, 1 GATA motif, 1 CTT motif, 1 GARE motif, 3 CGTCA motifs and 3 ABREs. The important *cis*-acting elements in the *EuPDF1B* gene promoter are listed in Table 1.

**Table 1:** Putative *cis*-acting regulatory elements identified in the promoter region of *EuPDF1B.*

*Cis* element	Sequence	Position	Function of site
ABRE	ACGTG	− 1606, − 1535, − 586	*Cis*-acting element involved in the abscisic acid responsiveness
G-box	TACGTG	− 586	*Cis*-acting regulatory element involved in light responsiveness
GT1-motif	GGTTAA	− 1601	Light responsive element
GATA-motif	GATAGGG	− 1246	Part of a light responsive element
CGTCA-motif	CGTCA	− 823, − 609, − 418	*Cis*-acting regulatory element involved in the MeJA-responsiveness
TCT-motif	TCTTAC	− 404	Part of a light responsive element
GARE-motif	TCTGTTG	− 136	Gibberellin-responsive element
chs-CMA1a	TTACTTAA	− 22	Part of a light responsive element
CAAT-box	CAAT, CAAAT	− 36, − 10	Common *cis*-acting element in promoter and enhancer regions
TATA-box	TATA	− 5	Core promoter element around -30 of transcription start

### Analysis of *EuPDF1B* promoter activity

**Figure 6:**
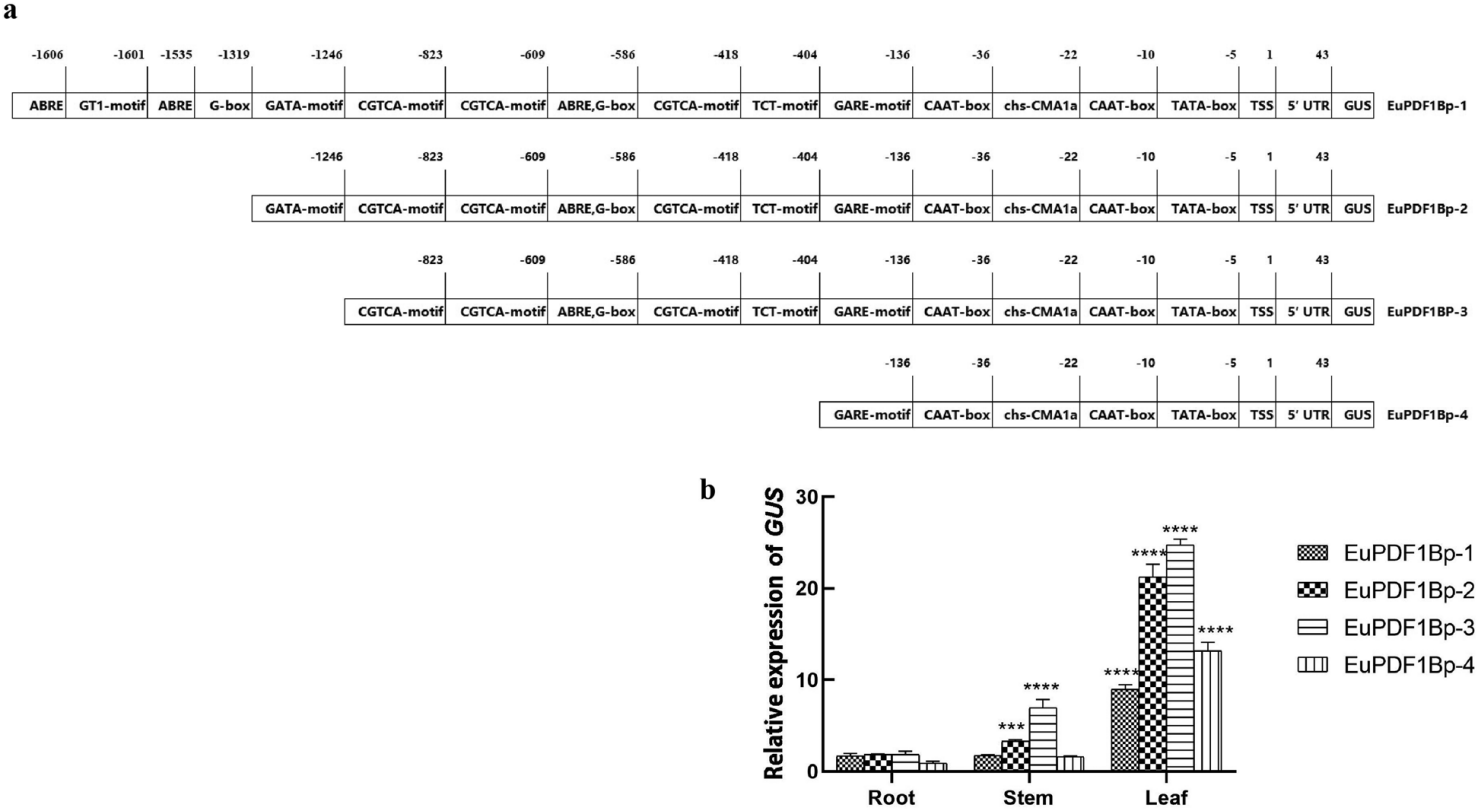
Analysis of *EuPDF1B* promoter activity. **a** Construction of *GUS* gene expression vectors driven by the *EuPDF1B* promoter with different length. **b** Relative expression levels of *GUS* driven by the *EuPDF1B* promoter with different length. The error bars represent standard deviations of three replicates, ***P < 0.001, ****P < *0.0001*.

**Figure 7:**
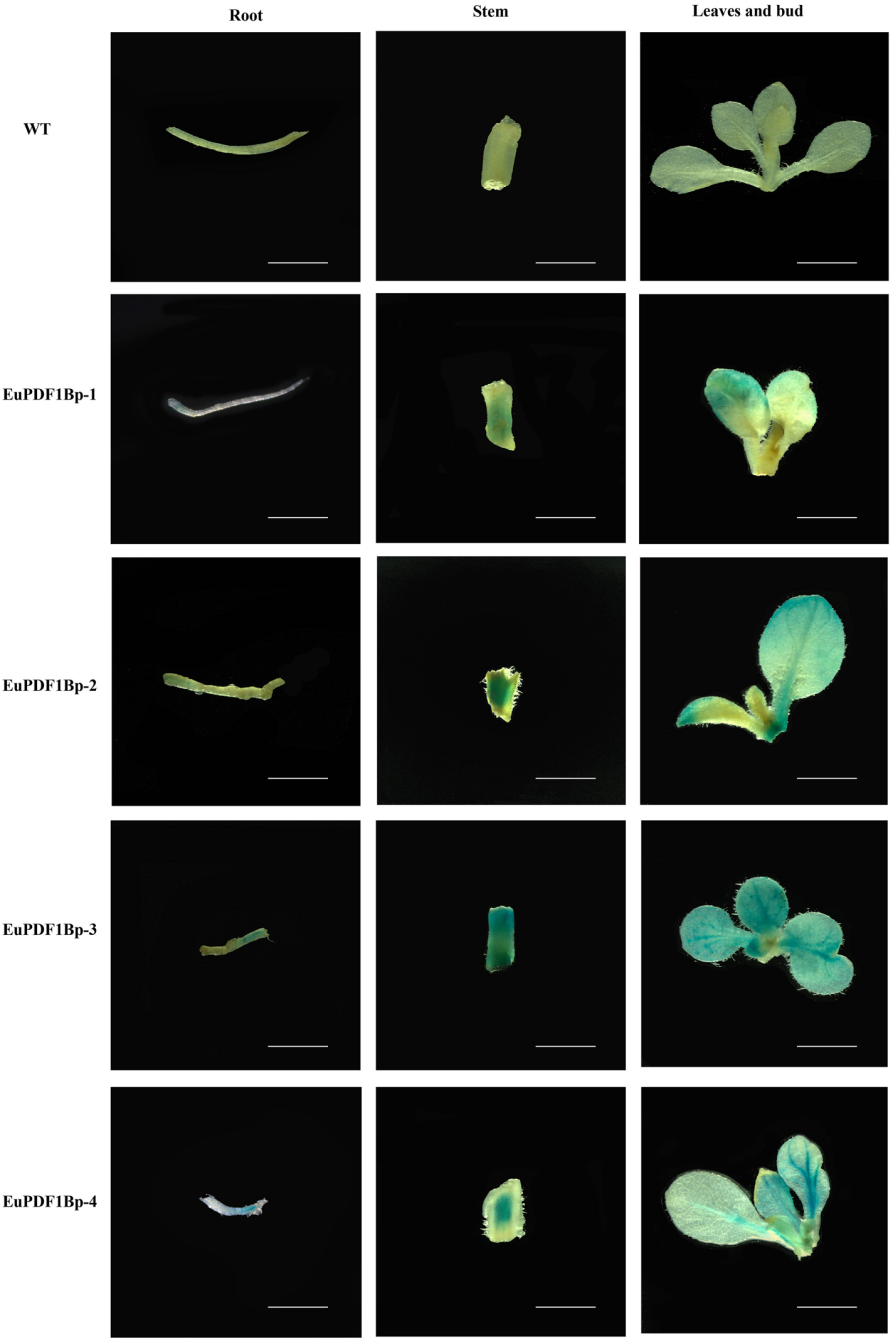
GUS protein expression in transgenic tobacco plants. Representative images showing GUS immunostaining of young shoots, roots, stems, leaves and buds of wild-type plants (WT), EuPDF1Bp-1, EuPDF1Bp-2, EuPDF1Bp-3, and EuPDF1Bp-4 transgenic plants. Scale bar = 3 mm.

To determine the activity of the *EuPDF1B* promoter, four plant expression vectors with the *GUS* reporter gene driven by the *EuPDF1B* promoter with different lengths and missing different response elements were constructed (Fig. 6a). Finally, transgenic tobacco plants were generated by incubating the leaf discs with the genetically engineered *Agrobacterium*. Histochemical GUS staining was performed on these transgenic tobacco plants, and the results showed that the wild-type tobacco plants did not stain blue, but all transgenic tobacco plants (EuPDF1Bp-1, EuPDF1Bp-2, EuPDF1Bp-3, EuPDF1Bp-4) stained blue (Fig. 7), indicating that promoters of all four lengths drove *GUS* reporter gene expression; that is, the core promoter of the *EuPDF1B* promoter was located in the − 236 bp region. The roots, stems and leaves of the transgenic tobacco plants were stained blue, but the blue colour in the leaves was deeper (Fig. 7), indicating that the *EuPDF1B* promoter mainly drives the expression of downstream genes in the leaves. The blue colour of EuPDF1Bp-3 and EuPDF1Bp-2 were relatively dark, and the blue colour of EuPDF1Bp-1 and EuPDF1Bp-4 were relatively light (Fig. 7), indicating that there may be enhancer elements in the − 891 bp to − 236 bp region of the *EuPDF1B* promoter. These results were confirmed by qRT‒PCR (Fig. 6b).

### Expression profiles of *EuPDF1B under* shading and hormone treatments

**Figure 8:**
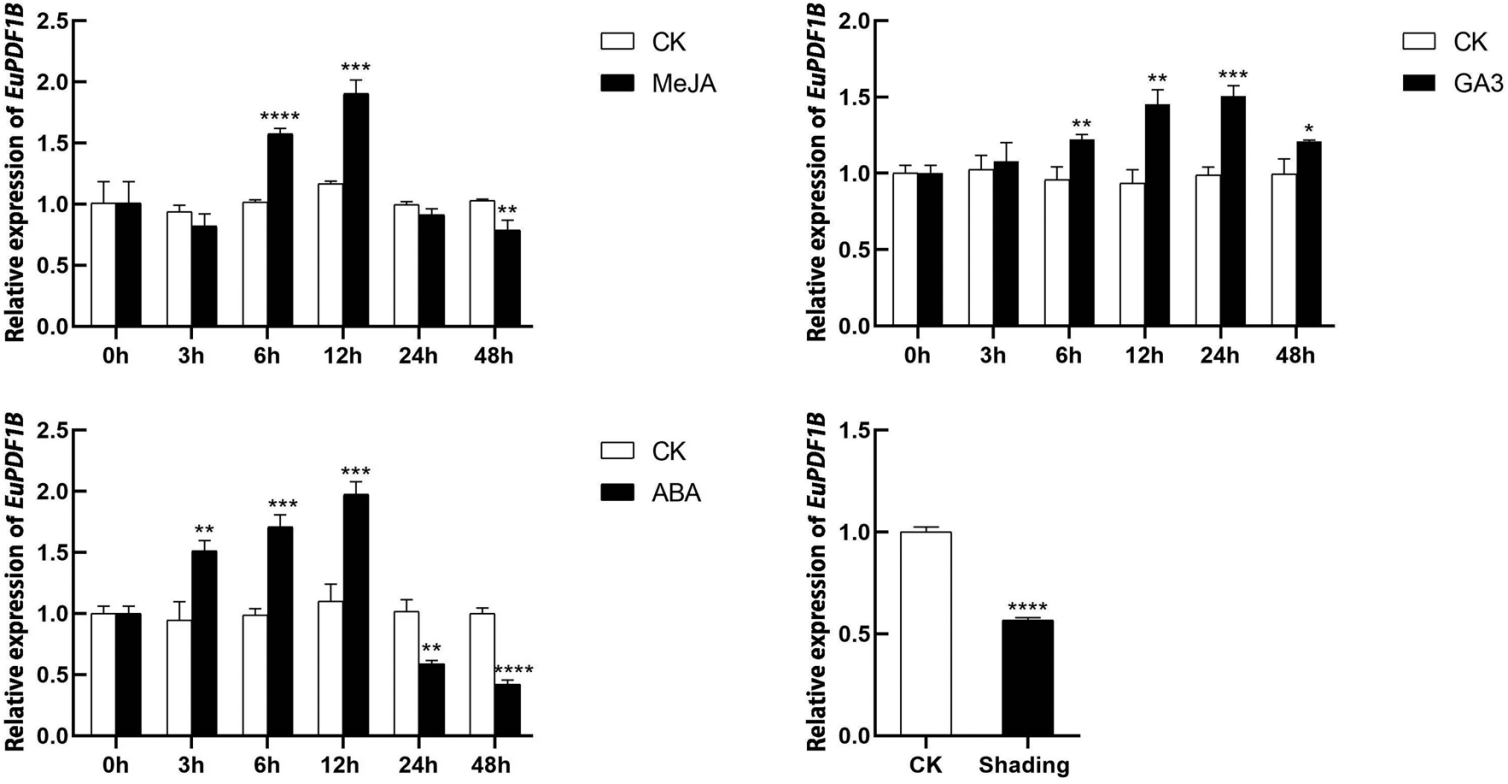
Relative expression levels of *EuPDF1B* in *E. ulmoides* seedlings under hormone and shading treatment. CK: negative control. The error bars represent standard deviations of three replicates, *P < 0.05, **P < 0.01, ***P < 0.001, ****P < *0.0001*.

Sequence analysis of the *EuPDF1B* promoter revealed photoresponsive elements, methyl jasmonate (MeJA) response elements, gibberellin (GA) response elements and abscisic acid (ABA) response elements. To verify whether light, MeJA, GA and ABA had any effect on the expression of *EuPDF1B* in *E. ulmoides* seedlings, qRT‒PCR was used to measure the expression of *EuPDF1B* after GA_3_, ABA, MeJA and shading treatment. After ABA or MeJA treatment, the expression of *EuPDF1B* gradually increased, peaked at 12 h after treatment and then rapidly decreased to a lower level than that before treatment (Fig. 8). After GA_3_ treatment, the expression level of *EuPDF1B* increased slightly and was 1.5 times that before treatment at 24 h after treatment (Fig. 8). After 2 days of shade treatment, the expression level of *EuPDF1B* decreased and was only 0.5 times that of the control plants (Fig. 8). These results suggest that the expression of *EuPDF1B* is regulated by GA_3_, ABA, MeJA and light.

**Figure 9:**
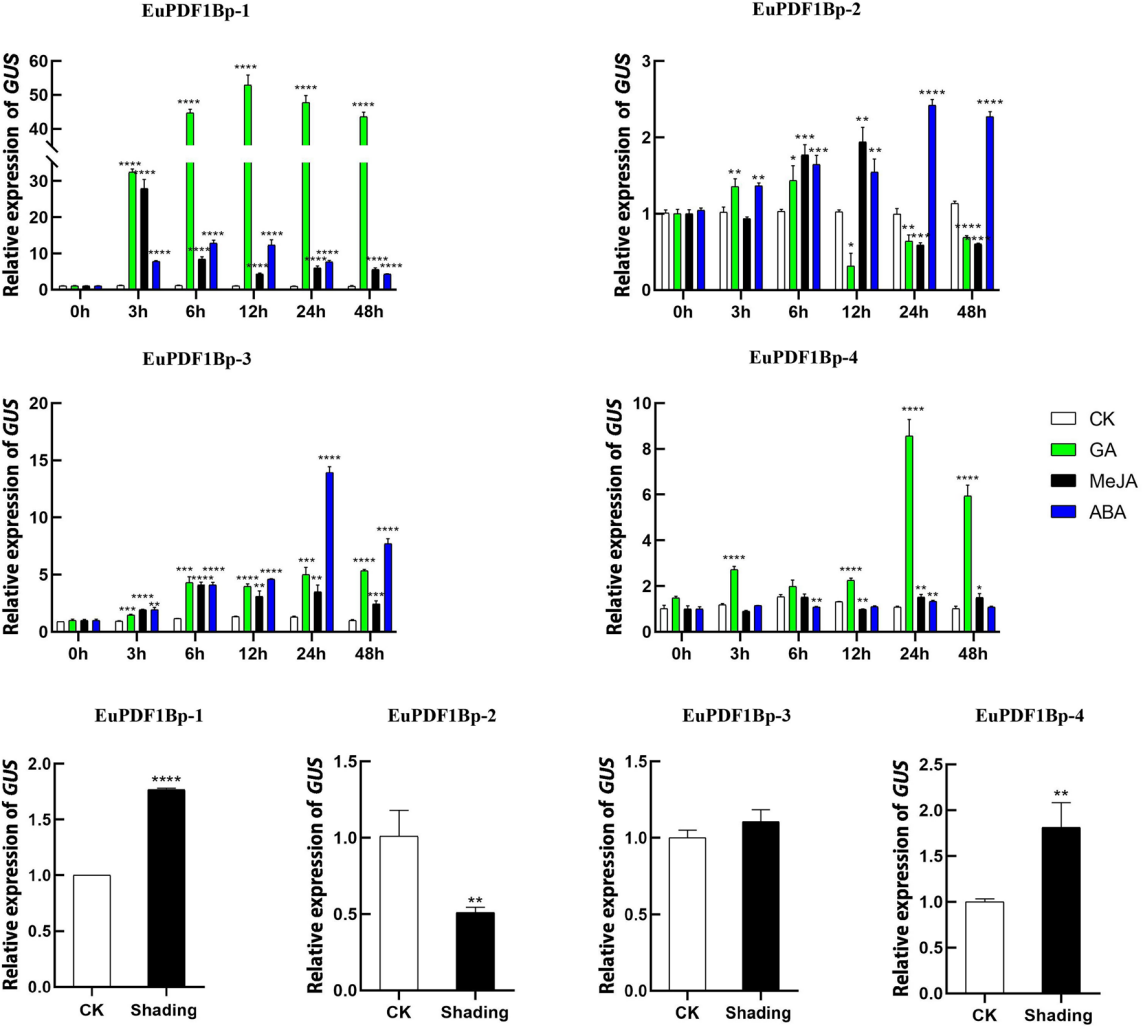
Relative expression of *GUS* under hormone and shading treatment. CK: negative control. The error bars represent standard deviations of three replicates, *P < 0.05, **P < 0.01, ***P < 0.001, ****P < *0.0001*.

To further determine whether light, MeJA, GA and ABA affect the expression of the *EuPDF1B* gene by affecting the activity of the *EuPDF1B* gene promoter, qRT‒PCR was used to determine the expression of *GUS* in the EuPDF1Bp-1, EuPDF1Bp-2, EuPDF1Bp-3 and EuPDF1Bp-4 transgenic tobacco plants after GA_3_, ABA, MeJA and shading treatment. GA_3_ induced *GUS* expression in the EuPDF1Bp-4, EuPDF1Bp-3, EuPDF1Bp-2 and EuPDF1Bp-1 plants. GA_3_, ABA and MeJA induced *GUS* expression in EuPDF1Bp-3, EuPDF1Bp-2 and EuPDF1Bp-1 plants, but the degree of *GUS* induction in EuPDF1Bp-2 was not as strong as that in EuPDF1Bp-1 and EuPDF1Bp-3. The expression level of *GUS* was increased in EuPDF1Bp-1 and EuPDF1Bp-4, decreased in EuPDF1Bp-2, and unchanged in EuPDF1Bp-3 after shading treatment (Fig. 9). These results were consistent with the predicted results of the promoter region, suggesting that GA, ABA and MeJA can promote the expression of *EuPDF1B* by increasing the activity of the *EuPDF1B* promoter, while low light can inhibit the expression of *EuPDF1B* by decreasing the activity of the *EuPDF1B* promoter.

## Discussion

The biosynthesis of all proteins begins with methionine. In the cytoplasm of eukaryotes, protein synthesis begins only with methionine, whereas in prokaryotes, as well as in the mitochondria and chloroplasts of eukaryotes, protein synthesis begins with N-formylmethionine^24^. The process of removing formyl groups from nascent peptide chains appears to be a conserved pathway in protein maturation. A mitochondrion-targeted PDF (PDF1A) and a chloroplast- and mitochondrion-targeted PDF (PDF1B) in higher and lower plants have been shown to be involved in the removal of the protein N-terminal formylmethionine in organelles^2,4^.

The retention of N-terminal formylmethionine in chloroplast-encoded proteins did not affect the accumulation of plastid RNA polymerase or plastid ribosome components but limited the accumulation of one or several PSII components and affected chloroplast PSII function^19^. Further studies have shown that treating tobacco seedlings with actinonin results in a rapid reduction in the accumulation of nascent D1 protein, which ultimately leads to the disintegration of the PSII complex, loss of PSII system activity, and leaf death^20^. Crystal structure and gel filtration chromatography evidence suggest that PDF1B is required in plants and has preferred substrate specificity for D1 polypeptides in PSII^23^. PSII in plant chloroplasts is essential for photosynthesis, accomplishing water photolysis and plastoquinone reduction by capturing light energy. As the photoreactive central protein of PSII, the D1 protein encoded by the chloroplast gene *psbA* plays a crucial role in the biogenesis and functional maintenance of PSII. Photosynthetic organisms encounter various abiotic stresses, such as low temperature, high temperature, and strong light, in their growth environment. The PSII complex of plants is the most vulnerable to damage from various abiotic stress processes^25,26^, but plants have evolved a repair process to renew damaged PSII complexes. This repair process involves the degradation and resynthesis of damaged proteins, particularly D1 proteins encoded by the chloroplast gene *psbA*, as D1 proteins are particularly vulnerable to damage during various abiotic stress processes^27^. Therefore, the rate of synthesis and degradation (or turnover) of D1 proteins represents a critical part of the PSII repair cycle. This cycle places the PSII complex in a photochemically active state^28^. Degradation of the damaged D1 protein begins with exposure to the N-terminus of the chloroplast matrix. Retention of the N-terminus formyl group of the D1 protein prevents the FtsH complex from recognizing the N-terminus of the D1 protein, thereby affecting the degradation of the D1 protein by the FtsH complex^29^. Blockage of D1 protein degradation affects the renewal and repair of damaged PSII. Since the degradation of the D1 protein during repair in PSII depends on the correct processing of its N-terminus, the removal of the N-terminal formylase by peptide deformylase is particularly important.

In transgenic tobacco plants overexpressing the *EuPDF1B* gene, the higher the expression level of *EuPDF1B*, the greater the expression level of *psbA*, indicating that the expression of *EuPDF1B* promotes the expression of the *psbA* gene. This finding is reasonable because the D1 protein encoded by *psbA* is the preferred substrate for the protein encoded by *EuPDF1B*. In addition, the height, fresh weight and net photosynthetic rate of the transgenic tobacco plants overexpressing *EuPDF1B* were greater than those of the wild-type tobacco plants. This may be because the overexpression of *EuPDF1B* promotes the expression of the *psbA* gene, which produces more D1 protein to participate in the assembly of PSII and timely replacement of the D1 protein damaged under abiotic stress, thus promoting the growth of tobacco plants. This finding is consistent with the observation that the chloroplast thylakoid membranes of transgenic tobacco plants overexpressing the *EuPDF1B* gene are more neatly arranged and densely packed than those of the wild type.

Moreover, it has been reported that the *PDF1B* gene of *A. thaliana* is involved in stress responses^1^. Therefore, it is speculated that PDF may play an active role in the process of plant resistance to abiotic stress. In our study, *EuPDF1B* expression was significantly induced by MeJA and ABA treatments. In addition, *cis*-acting element analysis of the *EuPDF1B* promoter revealed that it contains three ABA responsive elements (ABREs) and three MeJA responsive elements (CGTCA motifs), which respond to abiotic stress signals. This finding suggests that *EuPDF1B* may be involved in resistance to abiotic stress.

Histochemical GUS staining indicated that the *EuPDF1B* promoter mainly drives *GUS* gene expression in leaves. This result is consistent with the finding that *EuPDF1B* is expressed mainly in the leaves of *E. ulmoides.* In rice, the expression level of *PDF1B* in mature leaves was the highest among all tissues^22^. These results indicate that the expression patterns of *PDF1B* are similar in various plants. In this study, EuPDF1B was localized in chloroplasts but not in mitochondria, in contrast to the finding that PDF1B in rice and *S. lycopersicum* is located in both chloroplasts and mitochondria^22^. *EuPDF1B* is derived from the genome of *E. ulmoides.* There are differences in organ structure between woody plants and herbaceous plants, and the upstream regulatory sequences of the two plants are different, so the location of PDF1B may also be different. In addition, the expression level of *GUS* in EuPDF1Bp-3 and EuPDF1Bp-2 transgenic plants was significantly greater than that in EuPDF1Bp-1 and EuPDF1Bp-4 plants, indicating that enhancer elements were likely to be present in the − 891 bp ~ − 236 bp region of the *EuPDF1B* promoter. In our study, we identified and functionally characterized *EuPDF1B*, which laid a foundation for further analysis and study of *PDF* in woody plants.

## Materials and methods

### statement

None of the plants used in this study were wild and all the methods were complied with the IUCN Policy Statement on Research Involving Species at Risk of Extinction and the Convention on the Trade in Endangered Species of Wild Fauna and Flora.

### Plant materials, growing conditions and treatment methods

Mature seeds of *E. ulmoides* were collected from the Agricultural Biotechnology Base of Guizhou University. Wild-type tobacco seeds were preserved in laboratories. Both the male and female *E. ulmoides* plants used in the experiment were planted at the Agricultural Biotechnology Base of Guizhou University. Both the *E. ulmoides* seedlings and tobacco plants were grown under the following conditions: 16 h light/8 h dark photoperiod at 25 °C and 50% relative humidity. Fully developed tobacco leaves were used for genetic transformation tests. Leaves of 2-months-old *E. ulmoides* seedlings or transgenic tobacco plants were sprayed with 100 µM MeJA, 100 µM ABA or 100 µM GA_3_, and control plants were sprayed with an equal volume of solvent. The leaves were collected at 0, 3, 6, 12, 24 and 48 h after spraying. Leaves of 2-months-old *E. ulmoides* seedlings or transgenic tobacco plants were collected after 50% shade treatment for 2 days. All the samples were frozen in liquid nitrogen after collection and stored at − 80 °C until RNA isolation. qRT‒PCR was used to determine the expression level of *EuPDF1B* in *E. ulmoides* and the *GUS* reporter gene in tobacco plants.

### Cloning and sequence analysis of *EuPDF1B* and its promoter

After total RNA was extracted from *E. ulmoides*, reverse transcription reactions and 3′- and 5′-end amplification of the *EuPDF1B* gene were performed using a SMARTer RACE 5′/3′ kit (TaKaRa, Dalian, China). By sequencing and splicing the 3′ and 5′ ends of the *EuPDF1B* gene, a maximum ORF of 831 bp was obtained. Then, *EuPDF1B* was amplified by PCR, and the amplified product was constructed with the pMD18-T vector. Total DNA was extracted from the leaves using the CTAB method^30^. Then, specific primers were designed to amplify *EuPDF1B* promoter sequences of different lengths, and the cloned promoter fragments were constructed with the pMD18-T vector. The constructed vectors were subsequently sent to BGI for sequencing. The primers used are shown in Table 2. The CD-search tool in NCBI was used to predict the conserved domain of EuPDF1B, and the *EuPDF1B* promoter sequence of *E. ulmoides* was analysed by bioinformatics using the PlantCARE online website to identify possible regulatory motifs.

**Table 2:** Primers used for PCR.

Primer name	Primer sequence (5′–3′)*
RACE1	GATTACGCCAAGCTTGCCGGTTTCTCATGCTCTGCTGCCC
RACE2	GAATAGGTGCGGTGAA
RACE3	GCCAATAAGACTACACCG
RACE4	GAAAGGGCAGCAGAGCAT
F	GGTACCATGGCTTCTCCGACTTGGCT
R	GGATCCTCAGGATTTTCCAAAACCAACAGC
F1	GGTACCATGGCTTCTCCGACTT
R1	GTCGACGGATTTTCCAAAACCAAC
*EuPDF1Bp-*F1 (− 1714 bp)	GGAATTCCACGCGAACTGACATTTTGACC
*EuPDF1Bp*-F2 (− 1308 bp)	GGAATTCGTCGGGCACATCCTGACAAGT
*EuPDF1Bp*-F3 (− 891 bp)	GGAATTCGATTATGAAGCGGGGAGGGTCG
*EuPDF1Bp*-F4 (− 236 bp)	GGAATTCACCTGTCCACTCTAACCTGTC
*EuPDF1Bp*-R (43 bp)	CGGGATCCCACCCACAAGTGCAGTGGAGAA
*GUS*-F (qRT-PCR)	ACTGCTGCTGTCGGCTTTC
*GUS*-R (qRT-PCR)	GCACCTTGCGGACGGGTAT
*β-actin*-F (qRT-PCR)	GATCTTGCTGGTCGTGATCT
*β-actin*-R (qRT-PCR)	ACTTCCGGACATCTGAACCT
*EuPDF1B*-F (qRT-PCR)	CCTTTCTCAGTTGCCGGTGT
*EuPDF1B*-R (qRT-PCR)	TACAAATCGGCGGGAGAAGC
*EuActin*-F (qRT-PCR)	TTGTTAGCAACTGGGATGATATGG
*EuActin*-R (qRT-PCR)	CAGGGTGTTCTTCAGGAGCAA
*psbA*-F (qRT-PCR)	GCGACCTTGGATTGCTGTTG
*psbA*-R (qRT-PCR)	ATAGGGAGCCGCCGAATACA

*Underlined letters indicate the restriction enzyme sites

### Construction of the plant transformation vector and genetic transformation of tobacco

The maximum open reading frame sequence of *EuPDF1B* was inserted into the plant binary vector pCAMBIA1301 under the control of the CaMV35S promoter by the double enzyme digestion method to obtain the overexpression vector pCAMBIA1301-35S: *EuPDF1B*. *EuPDF1B* promoter DNA fragments of different lengths (1714 bp/1308 bp/891 bp/236 bp) were connected to the EcoRI and BamHI enzyme restriction sites of the pCAMBIA1381Z vector to obtain the following four vectors: pCAMBIA1381Z-*EuPDF1Bp*-1: *GUS* (1714 bp); pCAMBIA1381Z-*EuPDF1Bp*-2: *GUS* (1308 bp); pCAMBIA1381Z-EuPDF1B*p*-3: *GUS* (891 bp); pCAMBIA1381Z-*EuPDF1Bp*-4: *GUS* (236 bp). These vectors were used to transform *Agrobacterium* GV3101, which was subsequently genetically transformed into tobacco. PCR was used to identify the transgenic tobacco plants for further study. The primers used are shown in Table 2.

### Subcellular localization analysis of EuPDF1B

The maximum open reading frame sequence of *EuPDF1B* without a stop codon was inserted into the pCAMBIA1300-*GFP* vector to obtain the fusion expression vector pCAMBIA1300-35S: *EuPDF1B*-*GFP*. Moreover, the pCAMBIA1300-35S: *COX*-*RFP* vector was constructed to determine whether EuPDF1B was located in the mitochondria. The plasmids pCAMBIA1300-35S: *EuPDF1B*-*GFP* and pCAMBIA1300-35S: *COX*-*RFP* were simultaneously transformed into 3-week-old *A. thaliana* mesophyll cell protoplasts, while the plasmids pCAMBIA1300-35S: *GFP* and pCAMBIA1300-35S: *RFP* were transformed into 3-week-old *A. thaliana* mesophyll protoplasts as controls. Then, the protoplasts of *A. thaliana* were cultured overnight at 23 °C, and the fluorescence distribution was observed and photographed by laser confocal scanning microscopy.

### QRT‒PCR analysis

The accuracy of the RNA-seq results was verified by a qRT‒PCR system with RealStar Fast SYBR qPCR Mix (Genestar, Beijing, China). *β-Actin* and *EuActin*^31^ were used as internal reference genes for tobacco and *E. ulmoides*, respectively. The qRT‒PCR products were amplified as follows: 95 °C for 3 min, 40 cycles of 95 °C for 30 s, 60 °C for 30 s and 72 °C for 30 s. At least three independent biological replicates and three technical replicates were performed for each sample. The relative gene expression was calculated as described previously^32^.

### GUS histochemical staining analysis

The transgenic tobacco tissues were immersed in GUS staining solution and incubated at 37 °C for 24 h. The tissues were decolorized with 70% ethanol to completely remove chlorophyll and observed and photographed with a stereomicroscope (Nikon, Japan).

### Microscopic observation

The chloroplasts were analysed by transmission electron microscopy according to the methods described by Jung^21^. Each sample was washed with phosphate-buffered saline (pH 7.4) and fixed with 2.5% glutaraldehyde for 12 h, followed by 2% (w/v) osmium tetroxide for 2 h. After dehydration, the samples were sequentially embedded in EMbed 812 resin, polymerized into blocks after 3 days, and then sliced to 80 nm thickness by ultramicrosectioning. The chloroplasts were stained with 2% uranyl acetate and reynolds lead citrate for 10 min and 2 min, respectively, and then observed by transmission electron microscopy, after which the images were preserved.

### Statistical analysis

Statistical analyses were performed using SPSS software, and the significance of differences between the control and transgenic plants was analyzed using Student’s t-test. A* p* value of 0.05 was considered to be significant. Statistical significance was tested using Duncan’s test at 0.05 probability levels.

## Data Availability

The dataset used and/or analyzed in this study can be obtained from the author according to reasonable requirements. If someone wants to request the data from this study, please write to the author De-Gang Zhao at dgzhao@gzu.edu.cn.
